# RecGraph: recombination-aware alignment of sequences to variation graphs

**DOI:** 10.1093/bioinformatics/btae292

**Published:** 2024-04-27

**Authors:** Jorge Avila Cartes, Paola Bonizzoni, Simone Ciccolella, Gianluca Della Vedova, Luca Denti, Xavier Didelot, Davide Cesare Monti, Yuri Pirola

**Affiliations:** Department of Informatics, Systems and Communication, University of Milano – Bicocca. Viale Sarca 336, Milano 20126, Italy; Department of Informatics, Systems and Communication, University of Milano – Bicocca. Viale Sarca 336, Milano 20126, Italy; Department of Informatics, Systems and Communication, University of Milano – Bicocca. Viale Sarca 336, Milano 20126, Italy; Department of Informatics, Systems and Communication, University of Milano – Bicocca. Viale Sarca 336, Milano 20126, Italy; Department of Informatics, Systems and Communication, University of Milano – Bicocca. Viale Sarca 336, Milano 20126, Italy; Department of Statistics and School of Life Sciences, University of Warwick, Coventry CV4 7AL, United Kingdom; Department of Informatics, Systems and Communication, University of Milano – Bicocca. Viale Sarca 336, Milano 20126, Italy; Department of Informatics, Systems and Communication, University of Milano – Bicocca. Viale Sarca 336, Milano 20126, Italy

## Abstract

**Motivation:**

Bacterial genomes present more variability than human genomes, which requires important adjustments in computational tools that are developed for human data. In particular, bacteria exhibit a mosaic structure due to homologous recombinations, but this fact is not sufficiently captured by standard read mappers that align against linear reference genomes. The recent introduction of pangenomics provides some insights in that context, as a pangenome graph can represent the variability within a species. However, the concept of sequence-to-graph alignment that captures the presence of recombinations has not been previously investigated.

**Results:**

In this paper, we present the extension of the notion of sequence-to-graph alignment to a variation graph that incorporates a recombination, so that the latter are explicitly represented and evaluated in an alignment. Moreover, we present a dynamic programming approach for the special case where there is at most a recombination—we implement this case as RecGraph. From a modelling point of view, a recombination corresponds to identifying a new path of the variation graph, where the new arc is composed of two halves, each extracted from an original path, possibly joined by a new arc. Our experiments show that RecGraph accurately aligns simulated recombinant bacterial sequences that have at most a recombination, providing evidence for the presence of recombination events.

**Availability and implementation:**

Our implementation is open source and available at https://github.com/AlgoLab/RecGraph.

## 1 Introduction

Sequence-to-graph alignment is a fundamental computational problem in pangenomics (Computational Pan-Genomics Consortium 2018). Despite its relevance, some basic questions, such as what is a *good* sequence-to-graph alignment and what are its main desired features, have not been fully investigated from a theoretical viewpoint (Baaijens *et al.* 2022). Indeed, the focus so far has been on practical heuristics that can scale to population-scale human genomes and that are able to provide tools used by practitioners.

A seminal paper on the foundations of sequence-to-graph alignment is (Lee *et al.* 2002), where a notion of alignment of a sequence against a directed acyclic graph has been introduced. The resulting computational problem is the *partial order alignment* (POA). The original goal was to provide a practical solution to the multiple sequence alignment (MSA) problem, by iteratively adding sequences to the graph with a dynamic programming approach that extends the Needleman-Wunsch algorithm (Needleman and Wunsch 1970) to directed acyclic graphs. In fact, a sequence-to-graph alignment can be used to describe how to change the graph so that it is able to also express the sequence. While this paper predates computational pangenomics, POA has recently gained a renewed interest thanks to its ability to model and address the sequence-to-graph alignment problem. Some practical improvements have recently appeared, most notably abPOA (Gao *et al.* 2021), spoa, and Gwfa (Zhang *et al.* 2022) which incorporate recent advances in dynamic programming algorithms and SIMD instructions.

If the pangenome graph has cycles, the alignment problem becomes more complex and some formulations are NP-complete (Jain *et al.* 2020), for example when modifications in the graph are allowed to avoid mismatches. On the other hand, if changes are allowed only in the sequence, there is a O(|V| + q|E|)-time algorithm, where *q* is the length of the sequence and each vertex of the graph is labelled by a single character. Even outside computational pangenomics, approximate pattern matching in a graph has attracted interests, starting from (Amir *et al.* 2000, Navarro 2000) and going on with other important complexity results and algorithms for different variants (Thachuk 2013, Denti *et al.* 2018). More recently, the field has found new challenges and important contributions (Rautiainen and Marschall 2017, Garrison *et al.* 2018, Rautiainen *et al.* 2019, Sirén *et al.* 2021a,b).

The distinction between *variation graphs* and *sequence graphs* is crucial. In fact, variation graphs consider haplotype information represented as distinguished paths (Sirén *et al.* 2021a,b), while *sequence graphs* do not distinguish paths (Gao *et al.* 2021). On population-scale human pangenome graphs, the O(|V| + q|E|) time complexity of Jain *et al.* (2020) limits the practical usefulness of that approach; in fact heuristics are much more common on those data. Nevertheless, pangenomics is becoming relevant in the analysis of bacterial and viral species since the degree of variability in these species is even higher (Ding *et al.* 2018); while a in good quality alignment between two human genomes only 1% of its columns contain indels, when aligning two bacteria indels might appear in up to 50% of the columns (Colquhoun *et al.* 2021).

Bacteria frequently import genes, or fragments of them, in place of existing homologous genetic material in their genome, a process that was first identified by the observation of mosaic genes at loci encoding antigens or antibiotic resistance (Didelot and Maiden 2010, Yahara *et al.* 2014). These exchanges of material are known as homologous recombination (HG) and horizontal gene transfer (HGT). There have been significant efforts to understand and study bacterial pangenomes (Ding *et al.* 2018).

Pangenome graph structures for bacteria have been proposed recently (Colquhoun *et al.* 2021). Nevertheless, a recombination-aware alignment of a sequence against a pangenome graph has not yet been defined. Some initial efforts in that direction (Makinen and Valenzuela 2014, Rizzi *et al.* 2019) focus on pairwise alignments of diploid genomes. An important challenge is to construct a pangenome graph that expresses the mosaicism present in a species, such as the problem of discovering founders haplotypes (Bonnet *et al.* 2023), but that is not within the scope of our paper.

In this paper, we explore a first notion of sequence-to-graph alignment that exploits the fact that a pangenome graph represents a set of related individuals or species to highlight a phenomenon—in our case, recombination—that require a pangenomic (or population-based) approach for its detection. More precisely, we introduce the possibility that the sequence has been sampled from a genome that is the result of a recombination between two of the genomes represented in the graph. In our case, we explicitly model a recombination as a putative arc, and we describe a dynamic programming approach to compute an optimal sequence-to-graph alignment that allows a recombination, with a $O(|V{|}^{2}|\cdot n\;+\;P{|}^{2}\cdot |V|\;+\;|V|\cdot |P|\cdot n)$ time complexity, on a variation graph with |*V*| vertices, |*P*| paths, and a string with length *n*. The idea of adding a variation aspect to sequence-to-panel alignment is not entirely new. In fact, JALI (Spang *et al.* 2002) proposes a dynamic programming approach for aligning a query sequence against a multiple sequence alignment (MSA), where the alignment can switch between different panel sequences and discard some columns. Notice that this approach is not fully general, since for example it does not allow to jump backward in the MSA and thus it does not consider possible column duplications. Another relevant approach is Tesserae (Zilversmit *et al.* 2013), where an HMM approach to infer mosaic recombination is described together with the recurrence equation describing the Viterbi algorithm used to find the max likelihood path (Garimella *et al.* 2020).

We have implemented our approach in a tool called RecGraph, with an experimental analysis on bacterial graph pangenomes. Notice that, compared to state-of-art aligners to sequence graphs such as GraphAligner (Rautiainen and Marschall 2020), RecGraph allows alignments that would require the addition of new arc in the graph. Moreover, RecGraph takes into account in the alignment the cost of a recombination event, modelled as a linear function of the *displacement* of a recombination.

Even though a dynamic programming approach is unlikely to scale to genome-wide graphs, that is not a great limitation of our approach. In fact, almost all current alignment tools are based on a seed-and-extend strategy, where some exact (errorless) matches between the sequence and the graphs are first computed, those matches are chained, and finally the gaps within matches are filled in with a precise, but not necessarily fast, approach—e.g. Giraffe (Sirén *et al.* 2021a,b), a haplotype-aware sequence-to-graph aligner. Our main focus has been on this final task, where scalability to huge instances is not a requirement. Since recombination is particularly relevant in bacterial genomes, we explore the application of RecGraph in computing alignments of new recombinant bacterial sequences. Our main purpose is to assess the accuracy of the alignments produced by RecGraph in presence of novel recombinant genes. First we assess the high accuracy of alignments of RecGraph to variation graphs when allowing recombination. Secondly, using simulated recombination of the *slpA* gene of *Clostridioides difficile*, we show that RecGraph pangenome alignments achieve a sensitivity and specificity in reproducing the expected mosaic structure of recombinants that is close to 100%. In particular, our graph-based model of alignment recombination-aware achieves greater accuracy compared to JALI (Spang *et al.* 2002), a MSA-based competitive alignment model.

Finally, we assess the performance of RecGraph on real sequences of *C.difficile*; the phylogenetic trees confirm the predicted recombinant events. These results demonstrate that recombination-aware alignments against pangenome graphs open new perspectives in analysing bacterial genomes.

### 1.1 Preliminaries

Given an alphabet Σ, and s=s[1]⋯s[n] a string over Σ with length *n*, the substring *s*[*i*: *j*] denotes the portion of *s* from the *i*th character to the *j*th character, i.e. *s*[*i*: *j*] = *s*[*i*]⋯  *s*[*j*] The *k*-long prefix of *s*, i.e. the string *s*[1: *k*] is denoted as *s*[: *k*], while the *k*-long suffix of *s*, i.e. the string *s*[*n*-*k *+* *1: *n*] is denoted as s[n − k + 1:]. In this paper, we consider the notion of a variation graph that is a directed acyclic vertex-labelled graph, whose paths correspond to the genome sequences that we want to encode (Garrison *et al.* 2018, Baaijens *et al.* 2022). We refer the reader to Diestel (2005) for the terminology on graphs.

Definition 1 (Variation graph). A *variation graph* G=〈V,A,P,λ〉 is a directed acyclic graph whose vertices are labelled by nonempty strings, with $\lambda :V\to \Sigma^{*}$ being the labelling function, and where *A* denotes the set of arcs and *P* denotes a nonempty set of distinguished paths.

In the following we assume that a variation graph has a source node *s* and a sink node *t* such that all paths in *P* start in *s* and end in *t*. Moreover, the source and the sink are labelled by the empty string, since their only meaning is to quickly identify the boundaries of the genomes. In the literature, the notion of *sequence graph* is sometimes used: this corresponds to a variation graph where *P* consists of all possible source-to-sink walks (Baaijens *et al.* 2022). Given a variation graph G=〈V,A,P,λ〉 and one of its paths *p*, the *path label* of $p=\langle p_{1},\ldots ,p_{k}\rangle$ is the concatenation of the labels of the nodes in the path *p*, i.e. the string $\lambda (p_{1})\lambda (p_{2})\cdots \lambda (p_{k})$. With a slight abuse of language, we use λ(p) to denote the path label of the path *p*. Moreover, since we focus on acyclic variation graphs, there is no path of a variation graph G=〈V,A,P,λ〉 involving twice the same vertex. Notice that this constraint involves all possible paths of *G*, not only those in *P*. To simplify the presentation, we will only consider *canonical* variation graphs, where each vertex is labelled by a single character. It is possible to prove that considering only those graphs is not restrictive. In fact, some software tools available [e.g. abPOA (Gao *et al.* 2021)] convert the input graph into a canonical graph.

An alignment of a string against a graph is a sequence of pairs of positions of a path of the graph and of the string. Each pair can have an empty position, denoted with, in the case of a gap, but a pair cannot have both elements that are empty.

Definition 2 (Alignment of a string against a variation graph). Let G=〈V,A,P,λ〉 be a canonical variation graph, and let *s* be a string of length *l.* Then an alignment of *s* to *G* consists of *(1)* a path $p=\langle v_{1},\ldots v_{q}\rangle$ of *P* excluding the unlabelled source and sink of *G* and *(2)* a sequence $\langle (x_{i},y_{i})\rangle$ of *k* ordered pairs where each $x_{i}\in [1,q]\cup \{\;-\;\}$ and each $y_{i}\in [1,l]\cup \{\;-\;\}$ such that*:*
 

1. for any 1 ≤ i < j ≤ k such that both *x* ${\;}_{i}$, *x* ${\;}_{j}$ are different from *–*, then $x_{i}\;<\;x_{j}$*;*
2. for any 1 ≤ i < j ≤ k such that both *y* ${\;}_{i}$, *y* ${\;}_{j}$ are different from *–*, then $y_{i}\;<\;y_{j}$*;*
3. each pair has at least an element that is no*t –*, i.e. k ≤ q + l*;*
4. for each j∈[1,q] there is exactly one *i* such that $x_{i}=j$, and for each j∈[1,l] there is exactly one *i* such that $y_{i}=j$.

Informally, the alignment is specified by pairs $\left(x_{i},\;y_{i}\right)$ of positions of the alignment that are consistent with a left-to-right scan of both the string and the path *p* of the graph [conditions (1) and (2) of Definition 2]. More precisely, by condition (4) each vertex *v*${\;}_{j}$ of the path corresponds to exactly one position *x*${\;}_{i}$ of the alignment to which *v*${\;}_{j}$ is assigned, and similarly each symbol *s[j]* of the string *s*, is in exactly in a position *y*${\;}_{i}$ of the alignment. Pairs including symbol—correspond to the insertion of indels either in the path or in the string in the alignment. In particular, condition (3) corresponds to the usual requirement that no column of an alignment contains only indels.

Notice that Definition 2 describes a *global* alignment, but it can be extended to represent semi-global alignments that are more common when mapping reads—in this case we need to allow *p* to be a subpath of a path of *P*.

Given an alignment $\langle (x_{i},y_{i})\rangle$ with *z* ordered pairs, a *graph gap* consists of a maximal interval [b,e]⊆[1,z] such that all *x*${\;}_{i}$ with b ≤ i ≤ e are equal to –, while a *string gap* consists of a maximal interval [b,e]⊆[1,z] such that all *y*${\;}_{i}$ with b ≤ i ≤ e are equal to –. The *length* of such a gap is equal to e − b + 1, and will be denoted by *l*(*b*, *e*). In the following we will use the word *gap* to mean a string gap or a graph gap. The value of an alignment depends also on a score matrix *d* that assigns a value to each pair of characters, and a penalty for each gap of length *l*. In practice, we will consider only gap penalties that are proportional to its length, i.e. the penalty has the form g·l for a given constant *g*

Definition 3 (Value of an alignment). Let *s* be a sequence, let G=〈V,A,P,λ〉 be a variation graph, and let $p,\langle (x_{i},y_{i})\rangle$ be an alignment of *s* and *G.* Assume that $\langle (x_{i},y_{i})\rangle$ has *z* ordered pairs and *h* gaps $\left[b_{1},e_{1}],\ldots ,[b_{h},e_{h}\right]$, and let $B=\{j\in [1,z]:j\notin [b_{i},e_{i}]\;\text{for any}\;i\in [1,h]\}$*.* Then the *value* of the alignment is the sum

$$\sum_{i\in B}d(\lambda (p[x_{i}]),s[y_{i}])\;+\;\sum_{1\;\leq \;i\;\leq \;k}g(l(b_{i},e_{i})).$$

In Definition 3, p[k] is the *k*th vertex of the path *p* of the alignment, see Definition 2. The first component of the value is the sum of the values of all columns that are not part of a gap, computed by using the score matrix *d* while the second part is the sum of all gap penalties.

## 2 Materials and methods

To describe our method, we need to describe how to compute an optimal alignment without recombinations in a variation graph. We then formally define the concepts of displacement and alignment with a recombination (Section 2.1) and we propose an algorithm for computing such alignments (Section 2.2). We finally discuss (Supplementary Section S5.3) some possible improvements to avoid recomputing parts of the matrix when multiple paths share the same edge.

A trivial approach for computing an optimal alignment without recombinations in a variation graph is to extract the sequences corresponding to the paths and align the input string against each of those sequences (Needleman and Wunsch 1970, Marco-Sola *et al.* 2021), but this approach does not exploit the fact that the pangenome is stored as a graph. An alternative algorithmic approach that we adopt is extending the approach taken by POA (Lee *et al.* 2002) to the case of a variation graph G=〈V,A,P,λ〉. More precisely, POA (Lee *et al.* 2002) represents a Multiple Sequence Alignment (MSA) of a collection of sequences by a partial ordered graph, called PO-MSA, where nodes of the graph are single letters of the sequences and each sequence represents a path of the partial ordered graph. Then POA extends Smith-Waterman DP to the PO-MSA for aligning sequences to the acyclic graph.

Moreover, by Definition 2, the graph G=〈V,A,P,λ〉 has a nonempty set *P* of distinguished paths, among which we need to find a path p∈P that gives the optimal score in the global alignment of sequence *s* to *G*. Thus let *M[v*, *i*, *p]* be the optimal score of the global alignment between the initial portion of the path p∈P that ends in the vertex *v* and the *i*-long prefix *s*[: *i*] of sequence *s*. We can describe the values of the matrix *M* with the usual recurrence equation where, for simplicity, we denote with *g* the penalty of an indel, with *m* the score of a match, and with $\bar{m}$ the score of a mismatch:

$$\begin{array}{c} M[v,i,p]=\max\{\begin{array}{cc} M[u,i,p]\;+\;g & \left(1a\right)\\ M[v,i\;-\;1,p]\;+\;g & \left(1b\right)\\ M[u,i\;-\;1,p]\;+\;\bar{m}\;\text{if}\;\lambda (v)\neq s[i] & \left(1c\right)\\ M[u,i\;-\;1,p]\;+\;m\;\text{if}\;\lambda (v)=s[i], & \left(1d\right) \end{array} \end{array}$$

where λ(v) is the character labeling the vertex *v* and *u* is the vertex preceding *v* in *p*. Moreover, (i) $M[v_{0},0,\cdot ]=0$ if *v_0_* is the source of *G*, (ii) M[v,0,p]=M[u,0,p] + g if $v\neq v_{0},\;v\in p$, and *u* is the vertex preceding *v* in *p*, (iii) $M[v_{0},i,p]=ig$ if i > 0, (iv) M[v,i,p]= −∞ if v∉p. Notice that we are actually interested in $M[v_{m},|s|,p^{*}]$, that is the optimal global alignment of *s* with the path $p^{*}$ that maximizes $M[v_{m},|s|,p_{i}]$, for all paths $p_{i}\in P$, where *v*${\;}_{m}$ is the sink of the graph *G*.

### 2.1 Alignments with recombinations

We can now extend the notion of alignment to also allow recombinations. The main idea is that the result of a recombination is a mosaic of two subpaths, each extracted from a different path, of the variation graph *G*. The next step is to formally define a recombination.

Definition 4 (Recombination). Let G=〈V,A,P,λ〉 be a canonical variation graph. Then a recombination is a quadruple $\left(p_{1},p_{2},\rho ,\psi \right)$ where $p_{1}$ and $p_{2}$ are two different paths of G=〈V,A,P,λ〉, ρ, and ψ are two vertices, not necessarily distinct, respectively of $p_{1}$ and $p_{2}$, called *recombination vertices.*

By Definition 4, a recombination may induce a new path in the graph that connects the two paths *p_1_* and *p_2_* with the addition of a ‘virtual’ arc connecting the recombination vertices ρ and ψ. Alternatively, the new path is induced by switching from path *p_1_* to path *p_2_* in the recombination vertex ρ, when ρ=ψ.

In the following we define the alignment with a single recombination. The main intuition is that a high-quality alignment using a recombination is evidence that the variation graph *G* is lacking a path that is consistent with the sequence.

Using a single recombination means that the sequence and the graph are split into two parts: there is a standard alignment in between the first parts, another standard alignment between the second parts, and the recombination bridges the two parts. We can easily represent this bridge with a ordered pair of vertices.

Definition 5 (Alignment to a variation graph with a recombination). Let G=〈V,A,P,λ〉 be a canonical variation graph, and let *s* be a string with length *l.* Let (p,q,ρ,ψ) be a recombination of G=〈V,A,P,λ〉*.* Then an alignment of *s* to *G* with the recombination (p,q,ρ,ψ) is obtained from*:* (i) an integer 1 ≤ j ≤ l; (ii) subpaths *t_1_* of *p* and *t_2_* of *q*, such that *t_1_* ends in ρ and *t_2_* starts in ψ*.* The alignment consists of the concatenation of two alignments against a path: one between *t_1_* and *s[: j]*, and one between *t_2_* and s[j + 1:].

**Figure 1.**
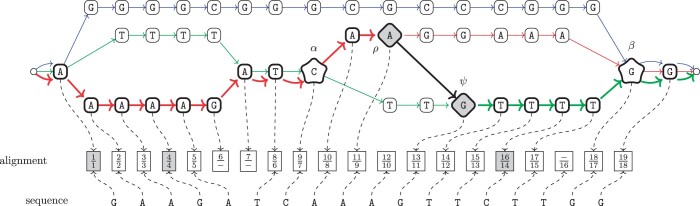
Example of alignment with a recombination of a sequence against a variation graph with three paths: *p_1_* is red, *p_2_* is green, and *p_3_* is blue. The recombination is represented by the thick black arc connecting two diamond-shaped vertices ρ and ψ with grey background. The branching vertex α and the consolidating vertex β are represented by stars. The nodes of the path *w* have thick edges. Pairs with a grey background correspond to mismatches.

In order to assign a value to an alignment with a recombination, however, the following definition of branching vertex and of consolidating vertex are needed to introduce the notion of displacement of a recombination: it will be instrumental in computing the penalty associated with a recombination. Notice that an alignment of a sequence with a recombination represents a new path not represented in the graph. Since the length of the new path may change with respect to the length of the two existing paths that are involved in the recombination, we need to assign a penalty to the choice of a recombination. Indeed, an arbitrary recombination may lead to a sub-optimal alignment when for example it consists of two paths and two recombination vertices that are quite apart from a the branching or consolidating vertex of the smallest bubble induced by the two paths in the variation graph. More precisely, given a recombination $\left(p_{1},p_{2},\rho ,\psi \right)$ on G=〈V,A,P,λ〉, there exist two vertices $\alpha_{p_{1},p_{2}}(\rho ,\psi )$ and $\beta_{p_{1},p_{2}}(\rho ,\psi )$, called respectively the *branching* vertex and the *consolidating* vertex of the recombination, such that (i) $\alpha_{p_{1},p_{2}}(\rho ,\psi )$ precedes ρ in *p_1_* and ψ in *p_2_*, and for each other vertex *v* that precedes vertex ρ on path *p_1_* and ψ on path *p_2_*, then *v* also precedes $\alpha_{p_{1},p_{2}}(\rho ,\psi )$, and (ii) ρ precedes $\beta_{p_{1},p_{2}}(\rho ,\psi )$ in *p_1_* and ψ precedes $\beta_{p_{1},p_{2}}(\rho ,\psi )$ in *p_2_*, and for each other vertex *v* such that is preceded by ρ in *p_1_* and by ψ in *p_2_*, then $\beta_{p_{1},p_{2}}(\rho ,\psi )$ also precedes *v*. Whenever the paths *p_1_, p_2_* and the vertices ρ, ψ are clear from the context, we will omit them, therefore using only α and β. The intuitive idea is that α and β are respectively the initial and final vertices of the smallest bubble of G=〈V,A,P,λ〉 including both ρ and ψ. Such two vertices always exist, since a variation graph has a distinguished source and a distinguished sink. See Fig. 1 for an example of an alignment and of nodes $\alpha =\alpha_{p_{1},p_{2}}(\rho ,\psi )$ and $\beta =\beta_{p_{1},p_{2}}(\rho ,\psi )$.

Just as a gap is more expensive the longer it is, we need to introduce a way to penalize differently recombinations connecting positions that are far away from each other, therefore implying a larger deletion or insertion. For this reason, we introduce the notion of displacement that is the analogous of gap length, where the length of a path is the number of its vertices.

Definition 6 (Displacement). Let $\left(p_{1},p_{2},\rho ,\psi \right)$ be a recombination. Let $\alpha =\alpha_{p_{1},p_{2}}(\rho ,\psi )$ and $\beta =\beta_{p_{1},p_{2}}(\rho ,\psi )$*.* Let *a_1_* be the subpath of $p_{1}$ from α to ρ*, b_1_* be the subpath of $p_{1}$ from ρ to β*, a_2_* be the subpath of $p_{2}$ from α to ψ*, b_2_* be the subpath of $p_{2}$ from ψ to β*.* Then the *displacement* of the recombination is $\left||a_{1}|\;-\;|a_{2}|\;+\;1\right|\;+\;\left||b_{1}|\;-\;|b_{2}|\;-\;1\right|$ and is denoted as $d_{p_{1},p_{2}}(\rho ,\psi )$.

The displacement of a recombination models how much the alignments are affected by the recombination and it is the difference of the distances of the vertices ρ and ψ respectively with α and β. Observe that the values + 1 and –1, added to $\left|a_{1}|\;-\;|a_{2}\right|$ and to $\left|b_{1}|\;-\;|b_{2}\right|$ in Definition 6 are due to the fact that ψ is the vertex following the vertex ρ in the recombination and a position is added to the right of ρ and subtracted w.r.t. ψ. Notice that we can compute and tabulate the displacement of all possible recombination in $O(|P{|}^{2}\cdot |V|)$ time by iterating over all pairs of paths and scanning in parallel those paths. Details are provided in the Supplementary Section. We focus our attention on a recombination penalty, defined by two parameters *d*${\;}_{o}$, called *opening recombination penalty* and *d*${\;}_{e}$, the *extending recombination penalty*, where the overall penalty is equal to $d_{o}\;+\;d_{r}\cdot d_{e}$, where *d*${\;}_{r}$ is the displacement of the recombination as defined in Definition 6.

### 2.2 The algorithm

We describe a dynamic programming approach for solving the following problem of computing a recombination-aware optimal alignment under an affine recombination penalty. Our approach is partly inspired by the linear-space dynamic programming algorithm for aligning two sequences (Hirschberg 1975) [also called forward-backward dynamic programming (Durbin *et al.* 1998)].

Problem 1 (Recombination-aware optimal alignment). *Given a variation graph* G=〈V,A,P,λ〉  *and a string s of length n, a score matrix d, a gap penalty g, and a recombination penalty* $\left(d_{o},\;d_{e}\right)$*, computes an alignment with optimal score to the variation graph with at most one recombination* (p,q,ρ,ψ)*, where p, q are two paths of the graph and* ρ  *and* ψ  *are the recombination vertices, with* ρ  *eventually equal to* ψ.

If the optimal alignment has no recombination, then we have already described the recurrence equation for the optimal solution. Alternatively, we run a complete forward pass and a complete backward pass, then we combine those results to place a recombination. More precisely, we define two DP matrices *M[v*, *i*, *p]* and *R[v*, *i*, *p]*, where: (i) *M[v*, *i*, *p]* is the optimal score of the global alignment between the initial portion of the path p∈P that ends in the vertex *v* and the *i*-long prefix *s*[: *i*] of the sequence *s* (forward pass); (ii) *R*[*v, i, p*] that is the optimal score of the global alignment between the final portion of the path p∈P that starts in the vertex *v* and the suffix *s*[*i* :] of the sequence *s* starting in position *i* (backward pass).

Notice that the coefficient *R[v*, *i*, *p]* is defined by reversing the direction of arcs in the graph *G* and considering the sink as the source of the graph, and vice versa; thus the recurrence for computing *R[v*, *i*, *p]* is exactly the same given for computing *M[v*, *i*, *p]* and has been given before as the Equation combining 1c, 1b, 1d, and 1a cases. Clearly, the two matrices can be computed in parallel. The value of an optimal alignment with at most one recombination between *s* and *G* is given by the following equation where *v_0_* and *v*${\;}_{m}$ respectively are the source and the sink of *G* and *n* is the length of the string *s*.

$$\begin{array}{c} \max\{\begin{array}{c} M[v_{m},n,p]\;\;\;\;\;\;\;\;\;\;\;\;\;\;\;\;\;\;\;\;\;\;\;\;\;\;\;\;\;\;\;\;\;\;\;\;\;\;\;\;\;\;\;\;\;\;\;\;\;\;\;\;\;\;\;\;\;\;\;\;\;\;\;\;\;\;\;\;\;\;\;\;\;\;\;\;\;\;\;\;\;\;(2a)\\ \underset{(v,w),j}{\max}M[v,j,p]\;+\;R[w,j\;+\;1,q]\;+\;d_{o}\;+\;d_{e}d_{\left(v,w\right)}(p,q),\;\;\;(2b) \end{array} \end{array}$$

where *p* and *q* are respectively the paths maximizing M[v,j,·] and R[w,j + 1,·]. If the maximum is achieved by case 2a, then the optimal alignment is obtained without any recombination. Otherwise there exists two paths p,q∈P, a position i ≤ n, and two nodes v,w∈V such that the optimal alignment of the sequence *s* against the graph *G* is made of the juxtaposition of the optimal alignment of the subpath of *p* from node *v_0_* to *v* against the prefix *s*[: *i*] of *s*, the recombination (p,q,v,w), and the optimal alignment of the subpath of *q* from node *w* to *v*${\;}_{m}$ against the suffix s[i + 1:] of *s*. Then the optimal score of the alignment is obtained by summing the optimal score of the recurrence *M* and *R* plus the displacement given by the value $d_{e}\cdot d_{\left(v,w\right)}$ meaning that the recombination corresponds to adding the virtual arc (*v*, *w*) if v≠w. As already observed, *v* and *w* might be the same vertex. Notice that we do not need to consider all possible paths. In fact, for each vertex *v* and position *i*, we only consider the path *p* maximizing *M[v*, *i*, *p]*, since any other choice would result in a suboptimal alignment. Symmetrically, for each vertex *w* and position *i *+* *1, we only consider the path *q* maximizing R[w,i + 1,q]. The time complexity of the naïve algorithm exploiting Equations (2a) and (2b) is $O(|V||P|n\;+\;|V{|}^{2}n\;+\;|P{|}^{2}n)$, since the algorithm computes the matrices *M* and *R* that have O(|V||P|n) cells, plus some additional data, in O(|V||P|n) time and space. In the same time, we can also determine, the paths maximizing *M[v*, *i*, *p]* and R[w,i + 1,q]. To compute the case 2b, we need to iterate over all possible pairs of vertices and all positions in the query sequence, therefore requiring $O(|V{|}^{2}n)$ time if we have precomputed all possible displacements which requires $O(|P{|}^{2}n)$ time (the details of the latter step are in the Supplementary Material).

## 3 Results

We implemented the method described in Section 2 in Rust and the tool, named RecGraph, is available at https://github.com/AlgoLab/RecGraph under the MIT license. RecGraph takes as input a graph in GFA format and a set of sequences in FASTA format and produces as output the optimal alignment of each sequence against the graph in the GAF format. We tested RecGraph in two different modes: in *no-recombination* mode it computes optimal alignments against a variation graph, while in *recombination mode*RecGraph computes optimal alignments against a variation graph and allowing (but not requiring) a recombination of two paths.

We designed an experimental evaluation divided in two parts, both inspired by the idea of exploring the efficacy and possible applications of an alignment to a graph that allows recombinations.

In the first experiment, we evaluated the quality of the alignments computed by RecGraph, measured by comparing the set of vertices involved in the alignment with the set of vertices of the true recombinant path.

In the second experiment, we focused on evaluating the accuracy of RecGraph in producing alignments that identify (i) the correct recombination breakpoint (by measuring the distance between the predicted) and (ii) the two paths among which the recombination took place.

All experiments were run on a 64 bit Linux (Kernel 5.15.0) system equipped with two AMD^®^ Epyc 7301 processors and 128 GB of RAM. The scripts, the data, and the instructions needed to reproduce the experiments are available at https://github.com/AlgoLab/RecGraph-exps.

### 3.1 Experiment 1: accuracy of graph alignments

We considered five bacterial species (Supplementary Table S1 in Supplementary Material) from the panX platform (Ding *et al.* 2018) and we simulated the scenario where a novel recombinant strain has to be aligned to the pangenome of some already-known strains. For each species, we randomly selected 100 genes whose length is between the first and third quartile (Supplementary Table S1 in Supplementary Material), and we created a directed acyclic sequence graph for each gene using the make_prg utility from Pandora (Colquhoun *et al.* 2021), which is the most widely used graph construction tool tailored for bacterial pangenomes. Since make_prg produces sequence graphs, in order to obtain a variation graph, we added the information of each input strain by tracing each path that corresponds to an input strain and assigning the proper sequence labelling. This results in a variation graph for each gene.

To guarantee that we have the ground truth (i.e. the correct path for each strain to align), we start from the variation graph *G*${\;}_{S}$ built on the set *S* of all the strains (paths), we identify a minimal set *K* of paths (‘known strains’) that cover all the edges of *G*${\;}_{S}$ and we remove the strains in S∖K from *G*${\;}_{S}$, obtaining the graph *G*${\;}_{K}$ that we have used in the experiment. Notice that *G*${\;}_{S}$ and *G*${\;}_{K}$ have the same vertices and edges since the paths corresponding to strains in *K* cover all the edges in *G*${\;}_{S}$, but they differ in their set of distinguished paths (see Definition 1). Set *K* is initially set to be equal to *S* and then each strain (path) p∈K is iteratively removed from *K* if path *p* does not cover (i.e. does not contain) at least an edge of *G*${\;}_{S}$ which is not covered by another strain of *K*. We finally compute the set R⊆S∖K of ‘recombinant strains’ such that *p*${\;}_{i}$ belongs to *R* if $p_{i}=p_{i}^{\prime }p_{i}^{\prime \prime }$ and $p_{i}^{\prime }$ and $p_{i}^{\prime \prime }$ are subpaths of two strains $s^{\prime }$ and $s^{\prime \prime }$ in *K*. Supplementary Table S1 (Supplementary Material) reports the number of recombinants simulated for each species. Additionally, we also altered each recombinant with different mutation rates (from 0% to 10%) to test the robustness of RecGraph in the presence of SNPs specific to the recombinant under analysis.

We aligned each recombinant strain using RecGraph to *G*${\;}_{K}$ in no-recombination mode and in recombination mode. We ran RecGraph using a linear gap penalty model (match score: 2; mismatch penalty: 4; gap penalty: 4). When ran in recombination mode, we set the recombination cost to 4 and the displacement multiplier to 0.1. For a comparison, we also aligned each strain in *R* to *G*${\;}_{K}$ with GraphAligner (Rautiainen and Marschall 2020), a state-of-art tool for aligning sequences to a *sequence graph*. Since GraphAligner does not align to a variation graph, it cannot limit or penalize the number of the implied recombinations, nor it can find a recombination whose recombination vertices are not the endpoint of an arc of the graph. We also tried to align the recombinants with giraffe (Sirén *et al.* 2021a,b), whose behaviour should resemble that of RecGraph in no-recombination mode, but it crashed on all instances. We conjecture that it is due to the length of the input sequences that have to be aligned since giraffe is designed to map short-reads and not long sequences as it is in this case.

We evaluated the accuracy of the alignments using three measures. First, we evaluated the Jaccard similarity coefficient between the set *P* of nodes of the path of the recombinant strain and the set *A* of nodes of the computed alignment. As usual, the Jaccard similarity is computed as $\frac{\left|P\cap A\right|}{\left|P\cup A\right|}$. We then evaluated each alignment in terms of edit distance between the input recombinant and the sequence expressed by the path it has been aligned to. Finally, we computed the minimum number of recombinations that explain the alignment by tracking the path and computing the number of switches via dynamic programming.

**Figure 2.**
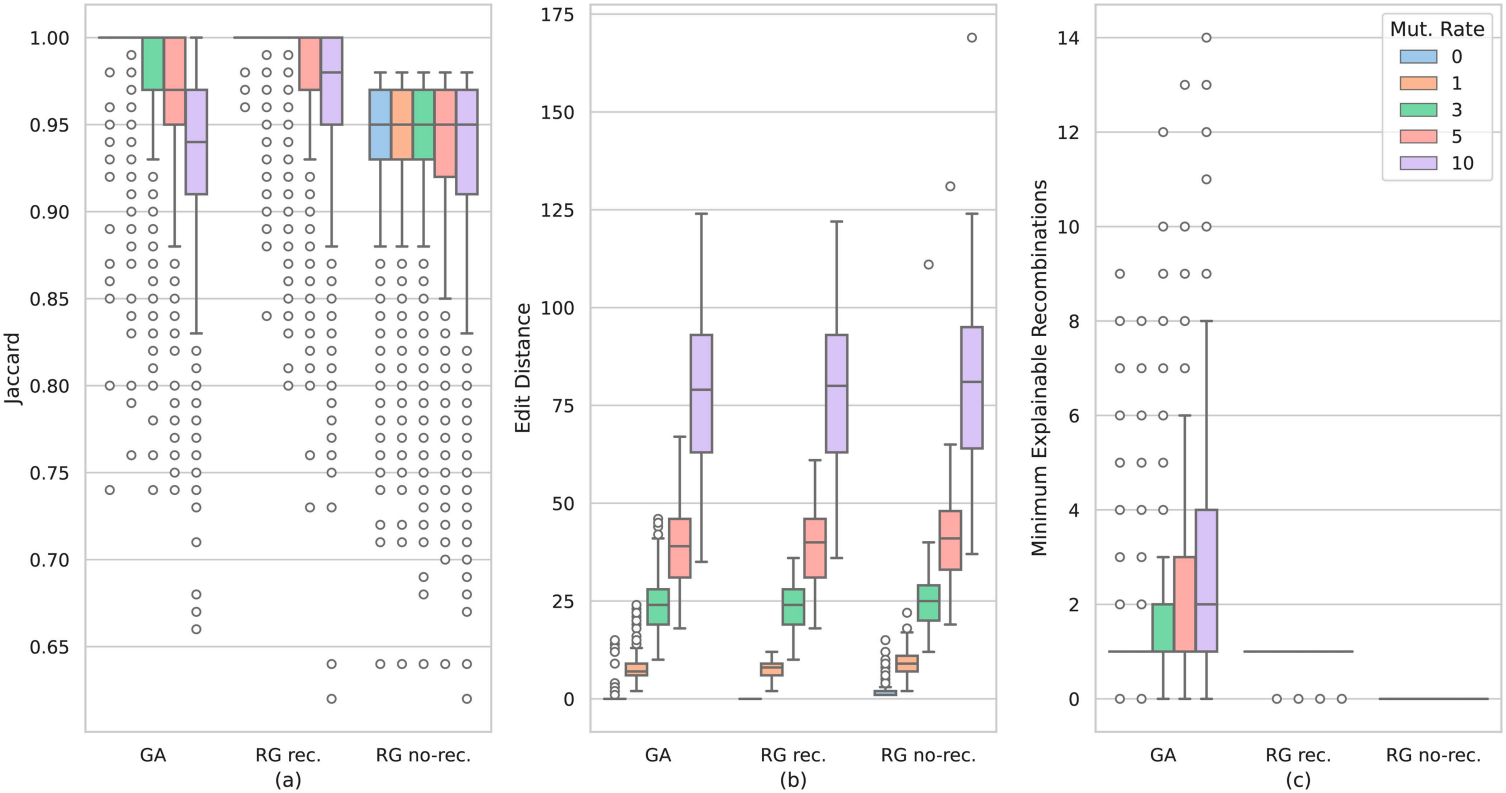
Results on alignments accuracy on simulated data. (a) Boxplot of Jaccard similarity coefficient between the real recombinant path and the path computed by RecGraph in no-recombination mode (RG no-rec.), GraphAligner (GA), and RecGraph in recombination mode (RG recomb.). (b) Boxplot of edit distance between the input recombinant sequence and the sequence spelled by the alignment path. (c) Boxplot of minimum number of recombinations explaining the alignment reported by the three tools. Boxplots are grouped by the mutation rate added to the input sequences (colors, from 0% to 10%).

Figure 2 shows the results of this analysis, grouped by simulated mutation rate (ranging from 0% to 10%). Figure 2a shows that, for all mutation rates, RecGraph in recombination mode computes alignments that are more similar to the true alignment than both GraphAligner and RecGraph in no-recombination mode. At mutation rate 0% (i.e. no mutations introduced in the recombinant strain sequences) both GraphAligner and RecGraph in recombination mode are able to perfectly reconstruct the path in at least the 75% of the cases. However, in 184 out of 3989 cases GraphAligner computes a sub-optimal alignment (Jaccard similarity between 0.01 and 0.98), while in 126 cases does not compute alignments at all. On the other hand, RecGraph always computed an alignment, and in all but six cases the alignment was correct. Manual inspection of those six suboptimal alignments revealed that they are due to repetitions in the labels of the vertices. As the mutation rate increases, we observe that the Jaccard similarity of both GraphAligner and RecGraph in recombination mode shifts towards lower values. However, the shift for GraphAligner is noticeable already at mutation rate 3% while for RecGraph in recombination mode the shift is noticeable only at mutation rate 5% or more. A more in-depth analysis shows that, as the mutation rate increases, GraphAligner computes alignments with slightly lower edit distance at the expense of implicitly introducing an increasing number of recombinations (on average, 1.04 at mutation rate 0% and 2.76 at mutation rate 10%—Fig. 2b). On the other hand, RecGraph in recombination mode exhibits on average a single recombination per alignment without being penalized in terms of edit distance whereas, as expected, RecGraph in no recombination mode never introduced a recombination at the expense of higher edit distance. A higher mutation rate resulted in higher edit distance for all tools. However, even though GraphAligner is free to introduce any number of recombinations in order to minimize the edit distance of its alignments, its accuracy in terms of edit distance is very similar to that of RecGraph in recombination mode. Remarkably, the edit distance of the alignments computed by RecGraph in recombination mode when mutation rate is low is better than GraphAligner edit distance. By manual inspecting these cases, we noticed that GraphAligner, due to its heuristic nature, struggles in correctly aligning a sequence in those regions of the graph comprising very short nodes on which it cannot easily place any anchor. RecGraph, instead, is able to compute an optimal solution even in those regions. On the other hand, RecGraph in no recombination mode exhibits higher edit distance in all cases (+1.5/2 on average, depending on the mutation rate). In contrast with other approaches that can introduce recombinations, RecGraph in no recombination mode is forced to align a sequence to a single path of the graph and cannot introduce any recombination to provide more accurate alignments (Fig. 2c). We recall, indeed, that we simulated data introducing a single recombination. Surprisingly, in 4.6% of the cases (the outliers in Fig. 2c), RecGraph in recombination mode reported no recombination. The number of alignments with no recombination increases as the mutation rate increases. Manual inspection of these cases revealed that, due to the chosen scores and penalties and the presence of mutations, staying on the same path while introducing the mismatches is more convenient than introducing a recombination. Indeed, when the mutation rate is 0, RecGraph in recombination mode always introduced a recombination. These results support our hypothesis that allowing a recombination in the alignment improves the accuracy of the computed alignments when the input sequences are recombinant compared to approaches where no recombination events are allowed (RecGraph in no-recombination mode being an optimal representative) while achieving very similar accuracy to approaches that do not exploit path information (e.g. GraphAligner) and producing more parsimonious results that are easier to evaluate.

RecGraph in recombination mode took from a few seconds to 3 min depending on the input graph size. We remind that our approach guarantees to find an optimal solution and that there are several heuristics that can be applied to speed up the computation—potentially forgoing this guarantee in a few cases. As expected, RecGraph in recombination mode is more time consuming than GraphAligner and its unrestricted and no-recombination counterparts, which took at most 10 s. All tested tools required less than 4 GB of memory.

### 3.2 Experiment 2: accuracy of recombination detection and breakpoint location

As opposed to the first experiment where recombinants are a result of a mosaicism inferred from the variation graph, in this experiment recombinants are simulated directly from real sequences. Indeed the main purpose is that of reproducing a recombination scenario in genes with high variability. Although the identification of recombinations is not the main goal of this work, RecGraph in recombination mode is applied here to evaluate its sensitiveness in locating the mosaic structure of simulated recombinants. More precisely, since recombinants are simulated from sequences, we have been able to more carefully evaluate the capability of RecGraph in aligning over a recombination breakpoint.

In *C.difficile*, the *slpA* gene encodes the main protein that constitutes the cell surface S-layer (Lanzoni-Mangutchi *et al.* 2022). This gene has been found to exhibit high diversity, with several clearly distinct variants identified in a phylogenetic study (Dingle *et al.* 2012). Starting from 11 such variants, we simulated a total of 994 new genes, each of which might be a recombinant of two random variants, with a randomly generated breakpoint. The location of the breakpoints was restricted to be between 10% and 90% of the sequence length since recombinations can be located if and only if both the prefix and the suffix contain at least a variation that distinguishes the two paths: if the prefix (or the suffix) is too short, then there is a significant probability that we do not have such a variation. Lastly, we randomly changed about 1% of the positions, therefore introducing single nucleotide variants only.

Each simulated sequence, of average length 2174 bp, was aligned using RecGraph, which took about 5 min per sequence, to the variation graph built from the 11 basis *slpA* sequences using make_prg. We ran RecGraph using a user-definable parameter to allow the introduction of a recombination only in the middle 95% of the input sequence. In this way we avoid to report alignment with recombinations that have little support in terms of variants that distinguish the two paths and, hence, that are likely false positives. Score for a match was set to two, while penalties were four for a mismatch and eight for a gap (linear gap penalty).

We remark that the aim of RecGraph is that of aligning while admitting and explicitly modelling the presence of a recombination: the actual prediction of recombinations is a downstream analysis effectively enabled by the alignments computed by RecGraph. For every simulated sequence generated without a recombination (*n *=* *102), RecGraph computed an alignment that does not include a recombination. Conversely, for every sequence simulated with a recombination (*n *=* *892), RecGraph computed an alignment exhibiting the correct recombination. Furthermore, the location of the breakpoint was estimated with high accuracy, with only on average 1.64 bp distance between the correct and inferred locations, with a standard deviation of 1.55 and 98% of the detected breakpoints within a distance of 5 bp.

To put results in perspective, we compared the accuracy of RecGraph with that of JALI (Spang *et al.* 2002), a tool that computes a ‘jumping alignment’ of a sequence against a multiple sequence alignment (MSA) of a set of sequences. A jumping alignment is an alignment of a given sequence against a given MSA of a set of sequences where, at each position (column), there is also the possibility to switch (jump) to another sequence (row) of the MSA. Hence the jumps could indirectly model the presence of recombination events. This part of the experiment aims to highlight the strengths and limitations of the MSA-based model compared to the graph-based model we propose. The comparison was performed on the set of 994 simulated sequences presented before. To ensure a fair comparison, we allowed RecGraph to place a recombination in the whole input sequence, rather than restricting it to the middle 95% of the sequence and we set the recombination extension penalty of RecGraph to $10^{\;-\;5}$ (effectively discarding the contribution of displacement). Score for a match of RecGraph and JALI was set to two, while penalty for a mismatch was four. JALI admits affine gap penalty and we used four for gap opening and two for gap extension penalties. RecGraph utilizes a linear gap penalty model with a penalty value of eight.

Table 1 reports the results obtained by varying how the input MSA was constructed from the 11 basis variants and by varying the recombination opening and gap extension penalties. The purpose is to assess how these choices affect the accuracy of the tools. Accuracy is evaluated by counting alignment errors. An alignment error is defined as a simulated sequence that is aligned with an ordered set of the 11 basis variants different from the one used for its simulation. In particular, JALI with gap extension penalty set to two exhibits low accuracy across all three MSAs and all choices for the recombination penalty (the number of errors ranges from 113 to 597 out of 994). We argue that this behaviour is mainly due to the inherent characteristics of having a linear ordering of the columns, as induced by the MSA: if the recombination implies a jump between rows of the MSA *and* skipping some columns, then the associated penalty could be greater than the penalties of various jumps between rows without introducing gaps. Indeed, the number of errors decreases as the recombination penalty increases. However, even with the largest recombination penalty (48), the number of errors remains high (113 out of 994 in the best case).

**Table 1.** Comparison of RecGraph and JALI.^a^^,^^b^

MSA	Tool	Gap ext. penalty	Recomb. penalty	No. of errors
Auto	JALI	2	4	511
			28	188
			40	145
			48	128
		0	4	189
			28	10
			40	4
			48	9
	RecGraph	2	4	14
			28	0
Sensitive	JALI	2	4	481
			28	176
			40	130
			48	113
		0	4	179
			28	16
			40	10
			48	12
	RecGraph	2	4	14
			28	0
gappy	JALI	2	4	597
			28	296
			40	243
			48	222
		0	4	269
			28	63
			40	42
			48	36
	RecGraph	2	4	14
			28	0

a The tools were run on 3 different MSAs (denoted with auto, sensitive, gappy) and using different scores for gap extension and recombination. Last column reports the number of computed alignments that do not match the basis sequences used for the simulation of the input sequences.

b MSAs were computed with mafft v7.520 with options: ‘auto’: —auto ‘sensitive’: —maxiterate 1000—globalpair—op 4—ep 2 ‘gappy’: —inputorder—anysymbol—allowshift—unalignlevel 0.8—leavegappyregion—maxiterate 2—retree 1—globalpair.

To further support our thesis, we ran JALI setting the gap extension penalty to 0 (essentially switching to a constant gap penalty, regardless of its length). With this choice, the accuracy of JALI significantly improves, especially with recombination penalties greater than the default (4). For example, the number of errors decreases by a factor of 18 for the first MSA with a recombination penalty set to 28 (from 188 errors to 10). When the gap extension penalty is fixed at 0, we can easily see that JALI is sensitive at how the input MSA is computed, even if the set of sequences on which the MSA has been computed does not change. Indeed, on average, best results were obtained on the ‘auto’ MSA, followed by ‘sensitive’, and ‘gappy’. Interestingly, in terms of the number of columns, the ‘auto’ MSA is neither the smallest (which is ‘sensitive’) nor the largest MSA (which is ‘gappy’), highlighting the difficulty of defining MSA characteristics that improve alignment accuracy.

Lastly, we observe that the choice of recombination penalty significantly influences the accuracy: small values allow to introduce many spurious recombinations to align the random mutations we introduced, while large values (e.g. 48) make JALI unable to introduce recombinations unless supported by several variants.

The extensive discussion we devoted to JALI accuracy contrasts with a straightforward analysis about RecGraph accuracy. Indeed, we can observe that the accuracy of RecGraph does not seem to be sensitive to the input MSA (we recall that the graph given as input to RecGraph is computed starting from the MSA) and that there is no need to tune the gap penalty to compute the correct alignments. Obviously, the recombination penalty plays an important role: a value of 4 allowed to introduce recombinations in 14 input sequences that were instead generated without recombinations. In these cases, the recombinations were introduced near the ends of the sequences and it sufficed to increase the recombination penalty to 28 (or to limit the placement of a recombination in the middle 95% of the sequence) to eliminate all spurious recombinations.

In conclusion, we argue that this experiment shows that the graph-based model of alignments accounting for a recombination achieves greater accuracy and is less sensitive to the choice of the parameters compared to a MSA-based (hence, linear-based) competitive alignment model.

### 3.3 Experiment 3: application to real data

Finally we tested RecGraph on a set of 265 real sequences of *C.difficile slpA* genes. Unlike the simulated sequences, there is no ground truth available on recombination events in the real sequences, thus rendering a quantitative analysis unfeasible. To assess the quality of the method we therefore run RecGraph and for each sequence in which a recombination is found we constructed two phylogenetic trees before and after the recombination point and checked the similarity of the partial recombinants to the two parts of each query sequence. RecGraph was run using a recombination cost of 28, while leaving all the other as default—i.e. match score of 2, mismatch penalty 4, gap open 4.

**Figure 3.**
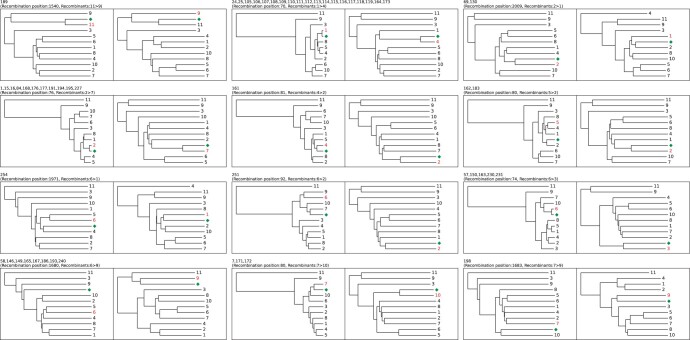
Trees computed before and after each recombination breakpoint found by RecGraph. The real sequences are aligned using clustalw, and phylogenetic tree were constructed before and after the breakpoint position using the UPGMA algorithm. Real sequences are clustered according to recombination paths and positions. In each pair of trees the cluster of sequences is indicated by the green diamond, and the recombinant sequences are highlighted in red.

Each possibly recombinant query is aligned to the 11 *slpA* basis variants using clustalw (Sievers *et al.* 2011), then two separate phylogenies are constructed before and after the recombination point reported by RecGraph using the UPGMA algorithm based on the pairwise distance matrix. Figure 3 shows the pairs of phylogenies constructed for the queries. For better visualization we clustered together queries expressing the same recombination breakpoint and recombinant basis variants. In each tree the query cluster is represented by a green diamond, while the recombinant basis variants are highlighted in red. In most of the cases the queries is closely related to the first recombinant variant in the first phylogeny, and to the second recombinant variant in the second phylogeny; thus supporting the recombination event claimed by RecGraph.

We run JALI on the same dataset using two set of configurations: default weights and optimized parameters chosen from the simulated experiment to obtain results most similar to RecGraph; respectively *gap extension* −2, *recombination cost* −4 (default) and *gap extension* 0, *recombination cost* −28 (optimized).

**Figure 4.**
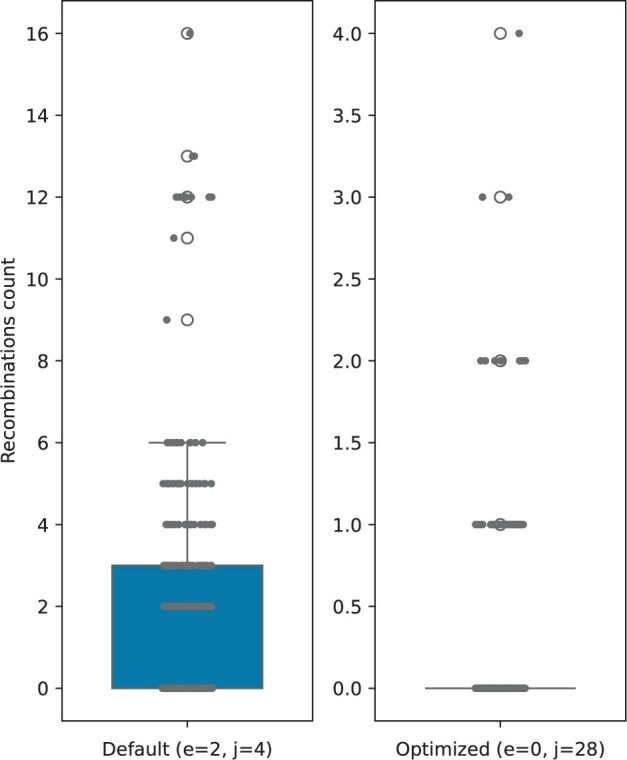
Number of recombinations found by JALI on the real dataset. (Left) JALI run with default parameters of gap extension (*e*) and recombination cost (*j*). (Right) JALI run with the optimized parameters chosen from the simulated experiment to obtain results most similar to RecGraph.

In Fig. 4 we show the total number of recombination events reported by JALI in both configurations; however due to the high number of such events it would be impossible to test the results using a similar phylogenetic approach as we did for RecGraph. Most notably, JALI is not designed to limit the number of recombinations, therefore in a substantial fraction of the cases it finds many putative recombinations. When we run it with its default parameters it finds up to 16 recombinations. This seems likely to be an overestimation since the *slpA* gene is only of length ∼2200 bp.

## 4 Discussion

We have extended the notion of aligning a sequence against a variation graph to also incorporate recombinations, which are subject to an affine penalty. We have developed RecGraph to compute sequence-to-graph alignments that allow a recombination, providing an experimental analysis that show that its alignments have higher quality when the sequence is the result of an actual recombination. This kind of alignment is especially relevant in bacteria that are characterized by a high degree of recombination events or horizontal gene transfer. In another experiment involving simulated recombinations, we showed that RecGraph can reconstruct recombination events with high accuracy, by correctly inferring the recombination breakpoints when such a recombination is present (or determining that the sequence is not involved in a recombination). While the main contribution of the present work is to present a recombination-aware sequence-to-graph aligner, detecting recombination in bacterial genomes is an important task that requires further developments of RecGraph. There are several reasons why detecting recombination is important in bacterial genomics. Firstly, recombination distorts the phylogenetic signal so that reconstructing a tree without accounting for recombination can lead to misleading results (Hedge and Wilson 2014). A direct application of RecGraph in this context would be to reconstruct separate phylogenies for each genomic regions separated by recombination breakpoints, the combination of which is equivalent to the concept of ancestral recombination graph (Didelot *et al.* 2010). Secondly, recombination events are often important evolutionary events, associated for example with adaptation (Sheppard *et al.* 2013), virulence (Wirth *et al.* 2006), and antibiotic resistance (Hanage *et al.* 2009, Perron *et al.* 2012). Thirdly, recombination has been shown to be linked with the concept of bacterial speciation (Falush *et al.* 2006, Fraser *et al.* 2007).

While our treatment of alignment is on variation graphs, it is immediate to extend our approach to sequence graphs—essentially we have to remove the path from the dynamic programming equation. In our opinion, such a modification is not very interesting, since we would not be able to limit the number of recombinations, but only the number of recombination arcs that do not appear in the original sequence graph, since the sequence graph does not contain the information associating a genome with a path.

A restriction of our problem is to compute an alignment against a variation graphs that exhibits no recombination. An exact dynamic programming formulation for this problem is in the Supplementary Material and is implemented in RecGraph. Notice that the time complexity this approach is smaller than the one of our main algorithm, since the absence of recombinations means that we do not compute an alignment on the suffix of the sequence.

An avenue for future research is the extension of RecGraph to longer genomes and sequences. In fact, almost all fully fledged aligners employ a seed-and-extend heuristics to quickly identify ‘easy’ parts of the alignment, where the sequence and the genome have a near-perfect match, then using a more refined, but slower, approach to fill in the gaps (Darling *et al.* 2010). RecGraph has been designed to work on those hard-to-solve parts of the alignment. A second direction is the integration of RecGraph in methods for the analysis of the mosaic structure of novel bacterial sequences by leveraging the properties of pangenome graphs. Indeed, this study is only the beginning of the investigation of the notion of pangenome graphs and sequence comparison to such graphs specialized for analysing high variable genes in bacteria. This can be thought of as an extension of previously described ‘copying models’ that have been popular to study recombination (Li and Stephens 2003, Yahara *et al.* 2014) which are unable to leverage alignments before analysis. In this direction, a future work will be the investigation of efficient algorithmic approaches that allow more than one recombination in the sequence-to-graph alignment.

There are also some possible future developments that mostly regard the algorithmic aspects of this paper. We can allow affine gap penalties in the alignment formulation, exploiting the technique described in (Gotoh 1982) to maintain the time complexity of our approach, but essentially requiring three times as much memory—a description is provided in the Supplementary Material. Another direction is to investigate the application of Hirschberg’s technique (Hirschberg 1975) to compute the *M* and *R* matrices. In this case, the challenge is to avoid making the tool too slow.

## Supplementary Material

btae292_Supplementary_Data
