# Supplementary material for: RecGraph: recombination-aware alignment of sequences to variation graphs
Source: Bioinformatics. 2024 Apr 27;40(5):btae292. doi: 10.1093/bioinformatics/btae292 (PMC11256948; doi:10.1093/bioinformatics/btae292)
Supplement: btae292_Supplementary_Data [file btae292_supplementary_data.pdf]

Supplementary material

5.1. Affine gap penalties

We describe how to extend our dynamic programming approach to allow affine gap penalites without increasing the overall time complexity, following the same ideas as (Gotoh, 1982).

Problem 2 (Recombination-aware optimal alignment) *Given a variation graph $G = \langle V, A, P, \lambda \rangle$ and a string s of length n, a score matrix d, an affine gap penalty g, and a recombination penalty (d_o, d_e), computes an alignment with optimal score to the variation graph with at most one recombination (p, q, ρ, ψ), where p, q are two paths of the graph and ρ and ψ are the recombination vertices, with ρ eventually equal to ψ.*

If the optimal alignment has no recombination, then we have already described the recurrence equation for the optimal solution. Alternatively, we run a complete forward pass and a complete backward pass, then we combine those results to place a recombination.

To compute the DP matrix $M[v, i, p]$, we also need to compute $E_s[v, i, p]$, $E_g[v, i, p]$, where: *(a)* $M[v, i, p]$ is the optimal score of the global alignment between the initial portion of the path $p \in P$ that ends in the vertex v and the i-long prefix $s[: i]$ of the sequence s (this definition is the same as in the main text); *(b)* $E_s[v, i, p]$ is the optimal score of the global alignment between the initial portion of the path $p \in P$ that ends in the vertex v and the i-long prefix $s[: i]$ of the sequence s, such that the alignment ends with the extension of a gap in the query sequence; *(c)* $E_g[v, i, p]$ is the optimal score of the global alignment between the initial portion of the path $p \in P$ that ends in the vertex v and the i-long prefix $s[: i]$ of the sequence s, such that the alignment ends with the extension of a gap in the graph.

The recurrence equations describing those matrices are the following where, for simplicity, we denote with g_o the gap opening penalty, with g_e the gap extension penalty, with m the score of a match, and with $\bar{m}$ the score of a mismatch:

$$M[v, i, p] = \max \begin{cases} M[u, i, p] + g_o & \text{(3a)} \\ E_s[u, i, p] + g_e & \text{(3b)} \\ M[v, i-1, p] + g_o & \text{(3c)} \\ E_g[v, i-1, p] + g_e & \text{(3d)} \\ M[u, i-1, p] + \bar{m} \text{ if } \lambda(v) \neq s[i] & \text{(3e)} \\ M[u, i-1, p] + m \text{ if } \lambda(v) = s[i], & \text{(3f)} \end{cases}$$

where $\lambda(v)$ is the character labeling the vertex v and u is the vertex preceding v in p. Moreover, (1) $M[v_0, 0, \cdot] = 0$ if v_0 is the source of G, (2) $M[v, 0, p] = \max\{M[u, 0, p] + g_o, E_s[u, 0, p] + g_e\}$ if $v \neq v_0$, $v \in p$, and u is the vertex preceding v in p, (3) $M[v_0, i, p] = g_o + (i-1)g_e$ if $i > 0$, (4) $M[v, i, p] = -\infty$ if $v \notin p$.

$$E_s[v, i, p] = \max \begin{cases} M[u, i, p] + g_o & \text{(4a)} \\ E_s[u, i, p] + g_e & \text{(4b)} \end{cases}$$

with $v \neq v_0$, $E_s[v_0, i, p] = -\infty$ if $i > 0$, and $E_s[v_0, 0, p] = 0$.

$$E_g[v, i, p] = \max \begin{cases} M[v, i-1, p] + g_o & \text{(5a)} \\ E_g[v, i-1, p] + g_e & \text{(5b)} \end{cases}$$

with $i > 0$, $E_g[v, 0, p] = -\infty$ if $v \neq v_0$, and $E_g[v_0, 0, p] = 0$.

A similar approach is used to compute $R[v, i, p]$, using the matrices $F_s[v, i, p]$, $F_g[v, i, p]$, where: *(a)* $R[v, i, p]$ is the optimal score of the global alignment between the final portion of the path $p \in P$ that begins with the vertex v and the suffix $s[: i]$ of the sequence s (this definition is the same as in the main text); *(b)* $F_s[v, i, p]$ is the optimal score of the global alignment between the final portion of the path $p \in P$ that begins with the vertex v and the suffix $s[i :]$ of the sequence s, such that the alignment begins with the extension of a gap in the query sequence; *(c)* $F_s[v, i, p]$ is the optimal score of the global alignment between the final portion of the path $p \in P$ that begins with the vertex v and the suffix $s[i :]$ of the sequence s, such that the alignment begins with the extension of a gap in the query sequence; the extension of a gap in the graph.

5.2. Computing the displacement

We describe here the procedure to efficiently compute the displacement of a recombination (p_1, p_2, ρ, ψ) where p_1 and p_2 are two different paths of $G = \langle V, A, P, \lambda \rangle$, ρ and ψ are two vertices, not necessarily distinct, respectively of p_1 and p_2. Moreover, let $T = \langle v_0, \ldots, v_z \rangle$ be a topological sort of $G = \langle V, A, P, \lambda \rangle$, that is $i < j$ for each arc (v_i, v_j), and v_0 (resp. v_z) is the source (resp. the sink) of $G = \langle V, A, P, \lambda \rangle$. Each path p is represented as an $|V|$-long bitvector $A[p]$ such that $AV[p][h] = 1$ iff the vertex v_h belongs to the path p.

Given the bitvectors $A[\cdot]$, for each pair (p_1, p_2) of paths we compute the bitvectors $BV[p_1, p_2]$ and $CV[p_1, p_2]$ containing respectively all branching vertices and all consolidating vertices of the two paths p_1 and p_2. In fact the vertex v_i is a branching vertex for p_1 and p_2 if and only if two conditions hold: (1) $AV[p_1][i] = AV[p_2][i] = 1$ (the vertex v_i belongs to both paths) and (2) $\text{select}(AV[p_1], \text{rank}(AV[p_1][i]) + 1) \neq \text{select}(AV[p_2], \text{rank}(AV[p_2][i]) + 1)$ (the vertex of that immediately follows v_i in p_1 is different from the vertex of that immediately follows v_i in p_2). A similar procedure computes $CV[p_1, p_2]$. Since each rank and select require constant time, the overall time complexity to compute all bitvectors $BV[\cdot, \cdot]$ and $CV[\cdot, \cdot]$ is $O(|P|^2|V|)$.

Then the branching vertex of the recombination (p_i, p_j, v_l, v_m) is the vertex v_h where h is the largest integer such that $BV[p_i, p_j][h] = 1$ and $h \leq i$, $h \leq j$, which can be computed in constant time via rank and select on BV. Computing the distance from v_h to v_l requires two selects on AV; in fact it is equal to $\text{select}(AV[p_1][l]) - \text{select}(AV[p_1][h])$. A similar idea allows to compute in constant time the distances from v_h to v_m and the distance from v_l (or v_m) to their consolidating vertex and consequently to compute the displacement of the recombination in constant time.

5.3. Possible improvements

In this section we will describe some ideas to speed up the computation of the matrix M (cases 1a, 1b, 1c, 1d). Let us first describe a possible improvement for RecGraph algorithm in mode *no recombination.* Let us recall that this mode is related to the basic equations used by RecGraph for detecting the optimal alignment of sequence against a specific path $p \in P$ of the variation graph, where P denotes a nonempty set of distinguished paths of the

graph. A main improvement is reducing the number of matrices that we need to fill, given k distinct paths in the graph. Indeed, we may fill a unique bidimensional matrix M^* with entries (v, i) and keep an additional matrix D to be accessed by using the entry v, i of M^* and a specific path denoted $\pi(v)$, called *representative path*, that is the path for which an optimal cost is achieved for the entry v, i.

The two matrices will replace the computation of matrix $M(v, i, p_j)$ for all paths $p_i \in Q$ crossing vertex v, which would require a $(|V| \cdot |P|n)$ time complexity. As detailed below, we are able to express the time with the new formula $O(|V| \cdot n + B \cdot |P| \cdot n)$, for B the number of times where we have a change of the representative path, given a position j on the query sequence, with $1 \leq j \leq n$.

The following proposition formalizes the main idea used for replacing the set Q of paths with a single representative path; it is a direct consequence of cases 1c and 1d where a gap is not introduced to compute the scores M.

Proposition 1 (Invariant scores) *Let p_1 and p_2 be two paths traversing the arc (u, v), and let $s[: i]$ be a prefix of the string s. Assume that the cases 1c, 1d that achieve the maximum for $M[v, i, p_1]$ are exactly those achieving the maximum for $M[v, i, p_2]$. Then $M[v, i, p_1] - M[v, i, p_2] = M[u, i-1, p_1] - M[u, i-1, p_2]$.*

Proof The Proposition is a consequence of cases 1c, 1d, as by these equations it holds that $M[w, j, 1] = M[u, j-1, p_1] + x$ and $M[w, j, p_2] = M[u, j-1, p_2] + x$ and thus the inequality holds in these cases. □

By Proposition 1, given p_1 and p_2 the two paths sharing the common edge (u, v), if no gap is introduced in position i then the differences $M[v, i, p_1] - M[u, i-1, p_1]$ and $M[v, i, p_2] - M[u, i-1, p_2]$ are the same. In other words, when computing $M[v, i, p_i]$ from $M[u, i-1, p_i]$, for every path $p_i \in Q \subseteq P$ sharing edge (u, v) we get the same increment.

The iterated applications of Proposition 1 is that whenever a subset $Q \subseteq P$ of all paths share a common subpath from u to v, we need to explicitly compute $M^*[u, i] = M[u, i, \pi(u)]$ for the representative path $\pi(u) \in Q$ along the path from u to v to get the optimal cost in the vertex v reaching j in the input sequence. Then given $M^*[v, j]$, we can obtain all other values $M[v, j, p_i]$, for any other $p_i \in Q$, by adding the difference $M[u, i, p_i] - M[u, i, \pi(u)]$, since that difference remains unchanged for all the vertices of the shared path from u to v. The difference from the optimal cost in vertex v of the value for path $p_i \in Q$, i.e $M[v, j, \pi(v)] - M[v, j, p_i] = D[v, p_i, j]$ is stored in memory in a vector D associated to v and a position j of the input sequence.

Thus only when the representative changes in a vertex v we need to update the vector D associated to v.

In other words the representative path $\pi(v)$ is the only path for which the score M is explicitly computed, for all other paths, when we need to have an update of the cost, due to a change of the representative, we need to consider vector D. Clearly, the computation of matrix M keeps associated the information represented by the pair $(\pi(v), v)$, i.e of the representative path and the vertex v, to which we associate the information D. Observe that the vector D may contain negative values. Now, we expect to compute the D vectors only for changes of the representative path and keep D updated for these changes. It follows that assuming that B is the number of times we change the representative path, then the computation of the costs for the paths is $O(B \cdot |P| \cdot n)$ which is summed up to the cost of computing M for a single representative path. Clearly, B in the worst case is $|V|$, but if the graph has many vertices of degree 2, then we expect to have a lower value for B. Indeed, each time we change the representative in a vertex u for a given position j over the query sequence, then $D(u, p_i, j)$ is updated for any path p_i crossing the same vertex of the representative path.

This fact can be exploited to speed up the computation of the optimal score M especially when the graph has a large number of non-branching paths, if no indel is introduced in aligning that portion of the path and the corresponding portion of the sequence.

To conclude the section, the discussed improvement can be applied in the implementation of the equation 2b.

Table S1. Information on bacteria species of Experiment 1. For each species, the table reports the number of strains, the total number of genes, the gene lengths (first quartile, average, and third quartile), and the number of recombinant strains extracted via our procedure.

Species	No. Strains	No. Genes	Lengths	No. Recomb.
E. albertii	19	2520	324/427/588	237
B. pseudomallei	63	3777	696/843/1006	525
S. pyogenes	125	996	681/828/977	874
P. aeruginosa	223	1932	624/777/918	1123
E. coli	500	732	564/720/861	1230

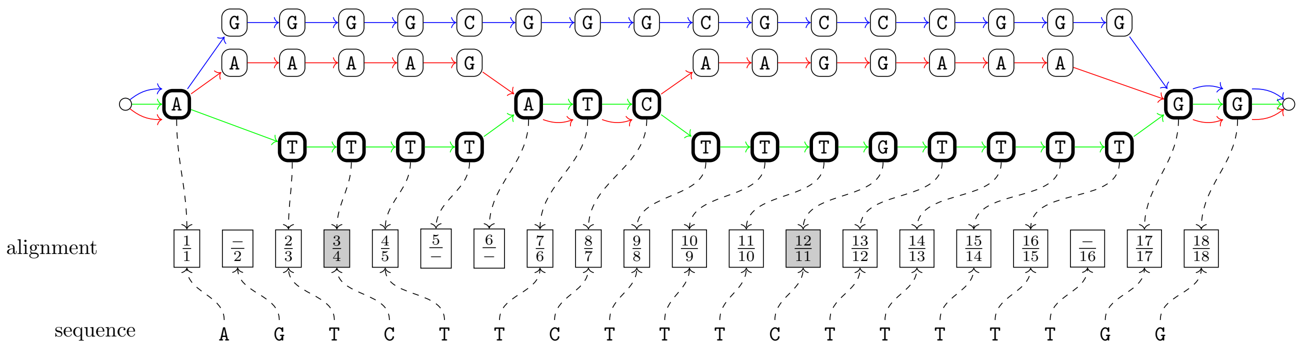

Figure S1: Example of no-recombination alignment of a sequence against a variation graph. The figure consists of three parts: the variation graph is above, the sequence is below, and a representation of the alignment is in the middle. The alignment consists of 20 pairs, where the number above is the position relative to the path of the graph and the number below is the position relative to the sequence. Moreover each pair is connected via a dotted arc to the character of the path and/or the character of the sequence that form the pair. This connection is inspired by the notion of threading scheme of (Maier, 1978). In the representation of the alignment, squares with a white background and no - represent a match, squares with a - represent an indel, and squares with grey background represent a mismatch.

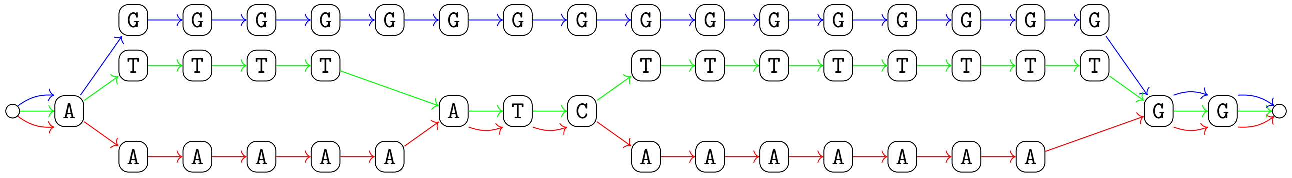

Figure S2: Example of canonical variation graph with three paths (one for each color).

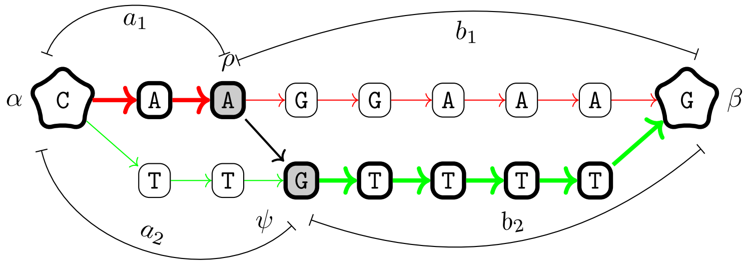

Figure S3: Displacement of the recombination in Figure 1. Only the portion of the graph between the branching vertex α and the consolidating vertex β nodes is represented. The recombination is represented by the thick black arc connecting the ρ and ψ vertices with gray background. The subpaths a_1, a_2, b_1, and b_2 are those of Definition 6. The displacement of the recombination is the sum of values $||a_1| - |a_2| + 1| = |3 - 4 + 1| = 0$ and $||b_1| - |b_2| - 1 = |7 - 6 - 1| = 0$, *i.e.* 0. Observe that a recombination may also use an existing arc in the graph in which case the arc represents a switch between two distinct colored paths.

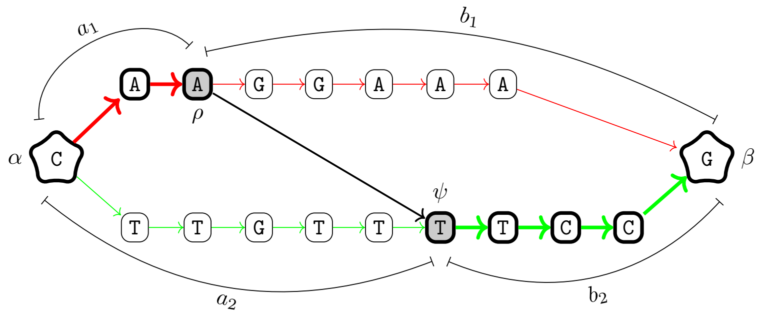

Figure S4: Example of recombination with a larger displacement. Only the portion of the graph between the branching vertex α and the consolidating vertex β nodes is represented. The recombination is represented by the thick black arc connecting the ρ and ψ vertices with gray background. The subpaths a_1, a_2, b_1, and b_2 are those of Definition 6. The displacement of the recombination is the sum of $||a_1| - |a_2| + 1| = |3 - 7 + 1| = 3$ and $||b_1| - |b_2| - 1 = |7 - 5 - 1| = 0$, *i.e.* 3.

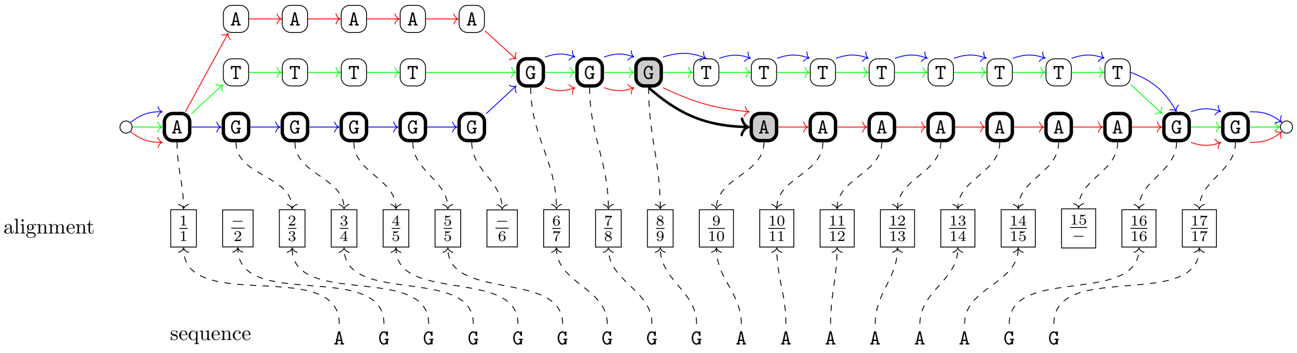

Figure S5: Example of recombination alignment of a sequence against a variation graph with a recombination. The recombination arc is represented by the thick black black arc connecting two vertices with gray background and is an arc already present in the graph. Thick vertices and arcs represents the portions of existing paths that form the alignment. In this case, the resulting alignment is a recombination between the red path and the green path.
